# Spatial and temporal variation in the abundance of *Culicoides* biting midges (Diptera: Ceratopogonidae) in nine European countries

**DOI:** 10.1186/s13071-018-2706-y

**Published:** 2018-02-27

**Authors:** Ana Carolina Cuéllar, Lene Jung Kjær, Carsten Kirkeby, Henrik Skovgard, Søren Achim Nielsen, Anders Stockmarr, Gunnar Andersson, Anders Lindstrom, Jan Chirico, Renke Lühken, Sonja Steinke, Ellen Kiel, Jörn Gethmann, Franz J. Conraths, Magdalena Larska, Inger Hamnes, Ståle Sviland, Petter Hopp, Katharina Brugger, Franz Rubel, Thomas Balenghien, Claire Garros, Ignace Rakotoarivony, Xavier Allène, Jonathan Lhoir, David Chavernac, Jean-Claude Delécolle, Bruno Mathieu, Delphine Delécolle, Marie-Laure Setier-Rio, Roger Venail, Bethsabée Scheid, Miguel Ángel Miranda Chueca, Carlos Barceló, Javier Lucientes, Rosa Estrada, Alexander Mathis, Wesley Tack, Rene Bødker

**Affiliations:** 1 Division for Diagnostics and Scientific Advice, National Veterinary Institute, Technical University of Denmark (DTU), Copenhagen, Denmark; 2 Department of Agroecology - Entomology and Plant Pathology, Aarhus University, Aarhus, Denmark; 3 Department of Science and Environment, Roskilde University, Roskilde, Denmark; 4 Department of Applied Mathematics and Computer Science, Technical University of Denmark (DTU), Copenhagen, Denmark; 5 National Veterinary Institute (SVA), Uppsala, Sweden; 6 Bernhard Nocht Institute for Tropical Medicine, WHO Collaborating Centre for Arbovirus and Hemorrhagic Fever Reference and Research National Reference Centre for Tropical Infectious Diseases, Hamburg, Germany; 7 Department of Biology and Environmental Sciences, Carl von Ossietzky University, Oldenburg, Germany; 8 Institute of Epidemiology, Friedrich Loeffler Institute, Greifswald, Germany; 9 Department of Virology, National Veterinary Research Institute, Pulawy, Poland; 10 Norwegian Veterinary Institute, Oslo, Norway; 11 Institute for Veterinary Public Health, Vetmeduni, Vienna, Austria; 12 CIRAD, UMR ASTRE, F-34398 Montpellier, France; 13 Institute of Parasitology and Tropical Pathology of Strasbourg, EA7292, Université de Strasbourg, Strasbourg, France; 14 EID Méditerranée, Montpellier, France; 15 Laboratory of Zoology, University of the Balearic Islands, Palma de Mallorca, Spain; 16 Department of Animal Pathology, University of Zaragoza, Zaragoza, Spain; 17 Institute of Parasitology, University of Zürich, Zürich, Switzerland; 18 Avia-GIS NV, Zoersel, Belgium

**Keywords:** *Culicoides* abundance, Seasonal abundance, Spatial pattern, Temporal trend, Vector season, *Culicoides* distribution, Europe, Vector-borne disease

## Abstract

**Background:**

Biting midges of the genus *Culicoides* (Diptera: Ceratopogonidae) are vectors of bluetongue virus (BTV), African horse sickness virus and Schmallenberg virus (SBV). Outbreaks of both BTV and SBV have affected large parts of Europe. The spread of these diseases depends largely on vector distribution and abundance. The aim of this analysis was to identify and quantify major spatial patterns and temporal trends in the distribution and seasonal variation of observed *Culicoides* abundance in nine countries in Europe.

**Methods:**

We gathered existing *Culicoides* data from Spain, France, Germany, Switzerland, Austria, Denmark, Sweden, Norway and Poland. In total, 31,429 *Culicoides* trap collections were available from 904 ruminant farms across these countries between 2007 and 2013.

**Results:**

The Obsoletus ensemble was distributed widely in Europe and accounted for 83% of all 8,842,998 *Culicoides* specimens in the dataset, with the highest mean monthly abundance recorded in France, Germany and southern Norway. The Pulicaris ensemble accounted for only 12% of the specimens and had a relatively southerly and easterly spatial distribution compared to the Obsoletus ensemble. *Culicoides imicola* Kieffer was only found in Spain and the southernmost part of France. There was a clear spatial trend in the accumulated annual abundance from southern to northern Europe, with the Obsoletus ensemble steadily increasing from 4000 per year in southern Europe to 500,000 in Scandinavia. The Pulicaris ensemble showed a very different pattern, with an increase in the accumulated annual abundance from 1600 in Spain, peaking at 41,000 in northern Germany and then decreasing again toward northern latitudes. For the two species ensembles and *C. imicola*, the season began between January and April, with later start dates and increasingly shorter vector seasons at more northerly latitudes.

**Conclusion:**

We present the first maps of seasonal *Culicoides* abundance in large parts of Europe covering a gradient from southern Spain to northern Scandinavia. The identified temporal trends and spatial patterns are useful for planning the allocation of resources for international prevention and surveillance programmes in the European Union.

**Electronic supplementary material:**

The online version of this article (10.1186/s13071-018-2706-y) contains supplementary material, which is available to authorized users.

## Background

Biting midges of the genus *Culicoides* (Diptera: Ceratopogonidae) are important vectors of viruses among livestock, for example bluetongue virus (BTV), African horse sickness virus (AHSV) and Schmallenberg virus (SBV). The incursion of these viruses in Europe in recent decades has caused substantial economic losses to farmers in the European Union [1–8].

At least 83 species of *Culicoides* are found in Europe (83 species reported in France [9]) but only some of these are suspected to transmit viruses: the afrotropical vector *C. imicola* was previously considered to be the main vector of BTV in southern European countries [10], though BTV virus was also isolated from wild specimens of *Culicoides obsoletus* (Meigen)/*Culicoides scoticus* Downes & Kettle and specimens of the Pulicaris ensemble [11–14]. During an unprecedented outbreak of BTV serotype 8 in northern Europe in 2006, it became evident that autochthonous *Culicoides* species of the subgenus *Avaritia*, specifically *C. obsoletus/C. scoticus* and possibly *Culicoides dewulfi* Goetghebuer, were transmitting the virus [15–19].

In 2000, the European Commission established a series of regulations for BTV control, including monitoring and surveillance in the affected countries. According to Commission Regulation (EC) No. 1266/2007, it is mandatory for member states to carry out bluetongue surveillance programmes that include vector monitoring [20]. As a result of the BTV outbreak in 2006, the northern European countries also started carrying out entomological surveillance of *Culicoides* vectors. Collections by light traps at ruminant farms have been a key component of these programmes in both southern and northern Europe.

Several European countries have gathered and analysed entomological data at a national level to determine the presence and abundance of different species of *Culicoides* [21–25]. In addition, vector activity during the winter has been assessed in an attempt to determine the existence of a “vector-free period” [26, 27]. Determining a vector-free period might be useful for national veterinary authorities to authorize movements of test-negative ruminants. The number of national studies from European countries has increased during the last decade, but the need to quantify *Culicoides* vector dynamics at a continental level remains, as European legislation for vector-borne diseases is founded on joint decisions among the member states.

The aim of the present study was to generate a joint entomological database for large parts of Europe comprising different climatic zones, using surveillance and research data collected during the period 2007–2013. Nine European countries (Spain, France, Germany, Austria, Switzerland, Denmark, Sweden, Norway and Poland) agreed to share data and quantify key temporal and largescale geographical trends in the abundance of two main *Culicoides* species ensembles (Obsoletus ensemble and Pulicaris ensemble) and *C. imicola.*

## Methods

### *Culicoides* database

**Fig. 1:**
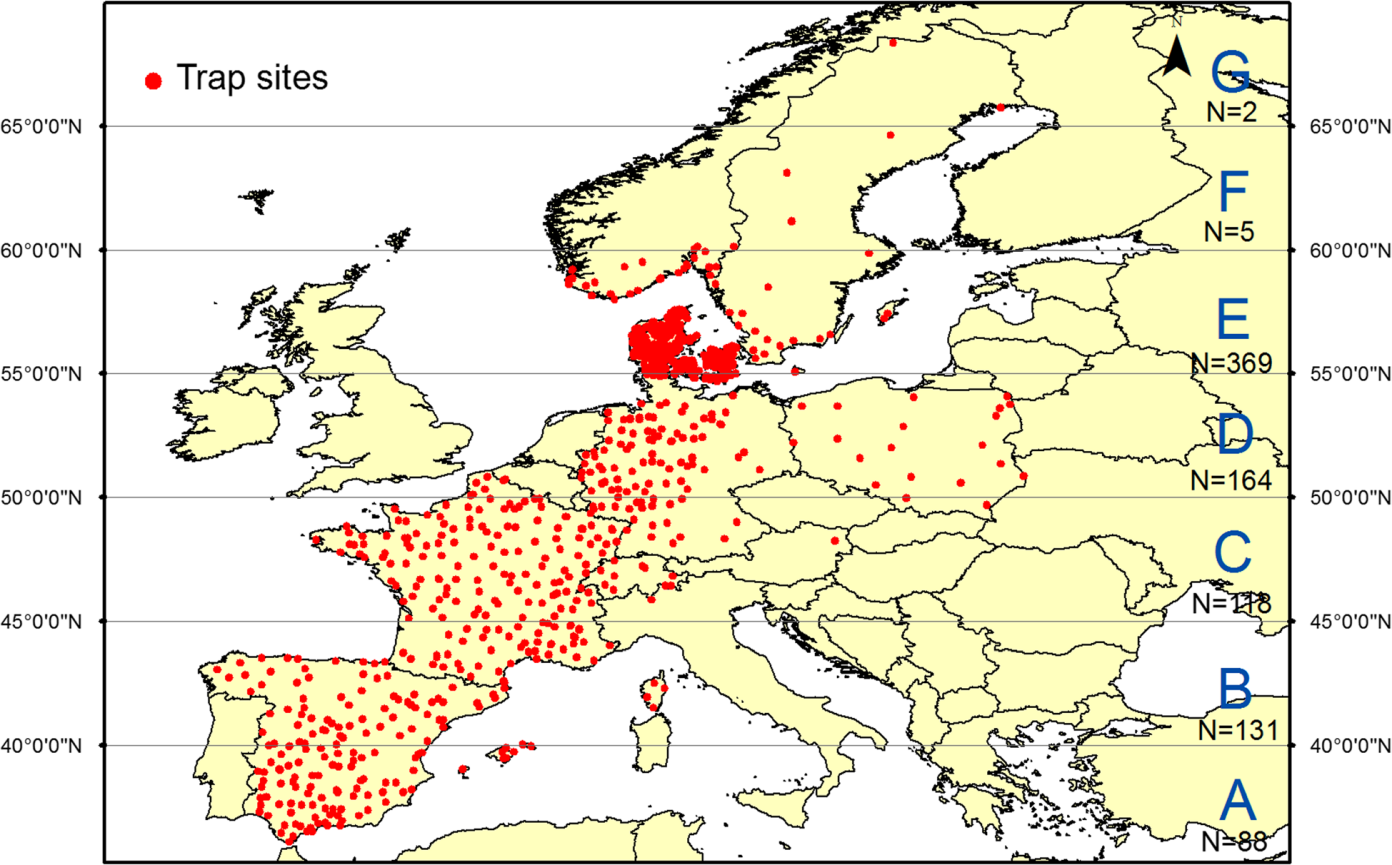
Available data from sampled farms in Europe during entomological surveys from 2007 to 2013. Latitudinal ranges were defined for every 5 degrees of latitude. From south to north, latitudinal ranges were named A, B, C, D, E, F and G

We gathered available *Culicoides* data from Spain [28], France [9, 23], Germany [24], Switzerland [29, 30], Austria [31], Denmark, Sweden [32, 33], Norway and Poland [34]. The data originated from national surveillance systems and research projects carried out during one or more years during a 7-year period (2007–2013) by national authorities and research groups. *Culicoides* biting midges were sampled from a total of 904 livestock farms (Fig. 1), with 31,429 trap collections comprising 8,842,998 specimens. For entomological sampling details from the different countries see [9, 23, 24, 28–34].

Black light suction traps were placed outside stables or near animal resting sites and the coordinates of each farm were recorded. Onderstepoort traps (Onderstepoort Veterinary Institute, Pretoria, Republic of South Africa) were used to catch *Culicoides* from dusk until dawn in all countries [23, 29, 31, 32, 34] except Germany, where Biogents Sentinel (BG-Sentinel) traps (BioGents, Regensburg, Germany) fitted with a black light lamp were used [24], and Spain, where mini CDC model 1212 (John W. Hock, Gainesville, FL, USA) traps were used [35]. Onderstepoort traps have been reported to catch more *Culicoides* than the other two types of traps [36, 37]. Therefore, data obtained by the BG-Sentinel and mini CDC traps were converted to the number of specimens estimated to have been collected if Onderstepoort traps had been used. For BG-sentinel traps, Venter et al. [36] calculated a conversion factor of 3.1, while Probst et al. [37] calculated a conversion factor of 3.83. We used the mean of these values (3.48) as the conversion factor in this study. Three trap efficiencies (0.404, 0.288 and 0.505) were previously reported for the CDC mini trap [36] .We used the average of these values (0.399) and used the reciprocal conversion factor of 2.51 for all vector species. A single trap per farm was used in all the countries with the exception of Germany. During the 2012–2013 campaign Germany operated 3 traps per farm, so in order to have only one observation per farm we took the median amount of *Culicoides* caught among the 3 traps.

Some *Culicoides* species are difficult to identify based on morphology, e.g. *C. obsoletus*/*C. scoticus* [13, 18, 38–41]. In the data available for this analysis, specimens were identified to species level in some countries, while other countries only identified them to group level. To create a uniform database, we aggregated the species into ensembles. We use the term “ensemble” to refer to a group of sympatric species for which morphological identification is sometimes not possible or difficult, and without phylogenetic meaning. In this work, “Obsoletus ensemble” refers to the Obsoletus group and *C. dewulfi* together and includes the following species: *C. obsoletus*, *C. scoticus*, *Culicoides montanus* Shakirzjanova, *Culicoides chiopterus* (Meigen) and *C. dewulfi*. The Pulicaris ensemble includes *Culicoides pulicaris* (Linnaeus) and *Culicoides punctatus* (Meigen). As it is commonly used in the literature while authors differ in its composition, we refer to the Obsoletus group as a species group including *C. obsoletus*, *C. scoticus*, *C. montanus* and *C. chiopterus*. Based on the phylogenetic analysis of Schwenkenbecher et al. [42], we considered *C. dewulfi* as a separate species from Obsoletus group. We focused on these seven species, as they are considered to be farm-associated species [27, 29]. *Culicoides imicola* specimens were identified to species level by the two countries in which they were found (Spain and France). The sampling period is shown in Additional file 1: Table S1 while the number of trapping farms per country, trap type, and national protocols of the specific country are presented in Table 1.

**Table 1:** Number of farms sampled, number of collections, trap type used, frequency of trapping, sampling protocol per country and number of *Culicoides* specimens trapped (without applying conversion factor)

National survey	Details	No. of farms sampled	No. of collections	Trap type	Frequency	Sampling protocol (nights)	Total no. of Obsoletus ensemble	Total no. of Pulicaris ensemble	Total no. of *C. imicola*	Total no. of *Culicoides*
Austria		1	1095	Onderstepoort	Daily	1	16,338	1888	0	18,226
Denmark 1	2008–2009	343	1087	Onderstepoort	1 per month	1	193,795	174,475	0	368,270
Denmark 2	2010 winter surveillance	31	233	Onderstepoort	1–5 per month	1	297	222	0	519
Denmark 3	2012–2013 summer surveillance	4	102	Onderstepoort	1–5 per month	2 (2013); 3 (2014)	79,796	61,121	0	140,917
Total Denmark		350	1422	Onderstepoort	1–5 per month	1–3	273,888	235,818	0	509,706
France		192	10,947	Onderstepoort	1–5 per month	1	3,728,710	154,742	258,904	3,728,710
Germany 1	2007–2008	89	1244	BG-Sentinel	Monthly	7	901,235	203,101	0	1,104,336
Germany 2	3 campaigns: Aug-Sep 2012, April-May 2013, June 2013	21	664	BG-Sentinel	1 per period	14	46,895	38,465	0	85,360
Total Germany		110	1908	BG-Sentinel		7 and 14	948,130	241,566	0	1,189,696
Norway		29	698	Onderstepoort	1–10 per month	1	1,274,685	77,662	0	1,352,347
Poland		19	559	Onderstepoort	Weekly	1	277,546	160,751	0	438,297
Spain		168	12,724	Mini CDC	1–14 per month	1	254,331	35,799	196,324	486,454
Sweden		23	363	Onderstepoort	Weekly	1	60,052	14,979	0	75,031
Switzerland		12	1713	Onderstepoort	Weekly	1–2	489,789	141,091	0	630,880
Total		904	31,429				7,323,469	1,064,299	455,228	8,842,998

### European temperature data

We obtained daily temperature data from Europe between 1994 and 2004 from MARS-Agri4cast. As previously described by Beek [43], these data resulted from a linear interpolation of weather stations distributed across Europe into regular climate grids of 25 × 25 km.

### Descriptive analysis and data management

We calculated the week number of each collection using the start date of the trapping. We defined week 1 of each year as 1st to 7th of January. This was done to ensure that the same dates from different years were given the same week number. We calculated the weekly and monthly mean abundance of vectors for each year. Finally, we calculated the average weekly and monthly abundance for the entire seven-year period to derive estimates for an “average year”.

We conducted three different analyses. In the first analysis, we divided Europe into seven latitudinal bands (A-G) of 5° width, from 35°N to 70°N (Fig. 1). Latitude range G (> 65 °N) contained only two farms with just 9 observations from 4 weeks in August and September 2008, and was therefore not included in the latitudinal range analysis. To compare the seasonal variation among the seven different latitude ranges, we log transformed the trap collection data [log_10_(x + 1)] and then calculated the mean of all the trap collections for each week number and at each latitude range, based on the data for the entire 7-year period (Fig. 1). To quantify the variation of the mean abundance for each week number, we calculated the 10th and 90th percentiles. For each latitudinal range, we also calculated the weekly average of the daily minimum, mean and maximum temperatures per week for the period from 1994 to 2004, and contrasted with the *Culicoides* seasonal variation. We estimated the number of vectors collected in an average year within each latitudinal range by calculating the annual cumulative sum of the weekly mean abundance and multiplying this by 7 days.

In the second analysis, we calculated the average abundance on each farm for each of the 12 months [log_10_(x + 1)]. We then spatially interpolated the log-transformed monthly averages to create spatial abundance maps. The interpolation was done using Inverse Squared Distance Weight (IDW) and based on the 15 nearest trap locations in ArcGIS 10.1 (ESRI, Redlands, CA, USA). We created buffer zones of 200 km around each farm and excluded all areas beyond this limit to avoid extrapolating to unsampled areas. In addition, countries outside the area of analysis were not included in the map.

In the third analysis we examined the spatial pattern of the start of the vector season by plotting the season start date of each NUTS (nomenclature of territorial units for statistics) 3rd level polygons defined by Eurostat (1992). NUTS is a hierarchical system used to divide up the economic territory of the EU for statistical purposes. We calculated the average start of the season for the 7-year period. The start of the season was defined as the first month in which the average monthly abundance per area polygon was equal to or higher than one specimen of *C. imicola,* and equal to or higher than 5 specimens of the Obsoletus and Pulicaris ensemble. The threshold numbers used here were based on (but not identical to) the threshold numbers defined by the European Commission to determine the start of the season [20]. While the EU thresholds are based on individual traps, we applied the thresholds to the average vector densities of all traps within a polygon. Polygons were classified as having “no data” if (i) the polygon did not have any sampled farms, (ii) if the mean abundance of the polygon did not reach the threshold during the year, or if (iii) there were no data available for the month prior the start of the season, thus making it impossible to detect whether the season might have started earlier.

## Results

The trap data made available for our analyses included a total of 8,842,998 biting midges that had been collected at 904 farms between 2007 and 2013 in nine European countries. Of these, 82.8% belonged to the Obsoletus ensemble, 12.0% to the Pulicaris ensemble and 5.1% were *C. imicola*. Biting midges of the Obsoletus and Pulicaris ensemble were found in all countries that were sampled, while *C. imicola* was only found in Spain, along the southern coast of France and in Corsica.

### *Culicoides* temporal fluctuation by latitude range

**Fig. 2:**
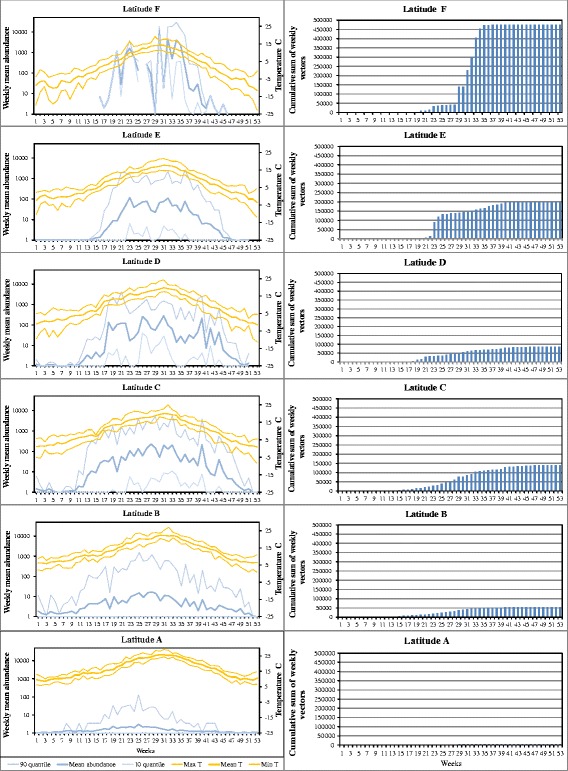
Left column: Obsoletus ensemble weekly average (log scale) with 10th and 90th percentiles for an average year per latitudinal zone (A-F). Right column: cumulative weekly number of vectors per year, by latitudinal zone. The latitudinal zones ranged from southern Spain (A) to the northern Scandinavia (F)

We observed a large variation in abundance in individual traps at each latitude range and at each week number, with the weekly 10th and 90th percentiles varying by a factor of 100 or more. However, when examining the average seasonal abundance, we were still able to observe two main patterns for the two species ensembles and *C. imicola*. First, the annual peak abundance in the Obsoletus ensemble increased gradually from Latitude A until it reached the highest weekly average peak of over 1000 vectors per night in Latitude F (Fig. 2, left column). Secondly, the period of the vector activity became increasingly shorter at higher latitudes, lasting throughout the whole year at Latitudes A and B (Fig. 2, left column), but only from week 15 (April) to week 46 (November) at Latitude F. Despite the increasingly shorter season for the Obsoletus ensemble further north, the cumulative sum of the weekly abundance steadily increased with latitude from less than 1000 Obsoletus ensemble vectors per year on average at Latitude A to 500,000 at Latitude F (Fig. 2, right column).

**Fig. 3:**
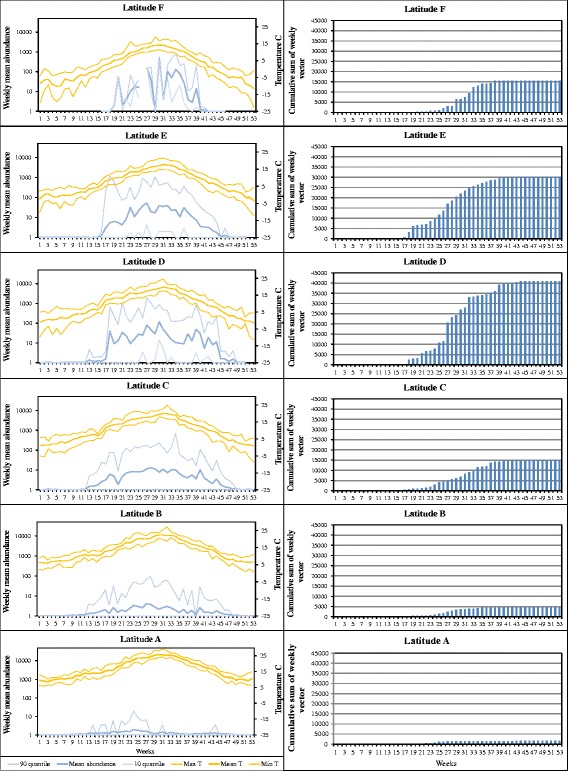
Left column: Pulicaris ensemble weekly average (log scale) with 10th and 90th percentiles for an average year per latitudinal zone (A-F). Right column: cumulative weekly number of vectors per year, by latitudinal zone. The latitudinal zones ranged from southern Spain (A) to the northern Scandinavia (F)

We found a similar trend in the abundance of the Pulicaris ensemble vectors from south to north: the weekly mean abundance increased gradually from less than 5 in Latitude A to a peak weekly average of 100 in Latitude D (Fig. 3). However, in contrast to the Obsoletus ensemble, the Pulicaris ensemble abundance did not continue to increase beyond Latitude range D. In Latitude F, there was a marked variation in abundance across weeks (varying from low abundance one week to high peaks the next week). The cumulative sum of the weekly mean of Pulicaris ensemble abundance showed a different pattern compared to the Obsoletus ensemble, with the mean accumulated number of Pulicaris ensemble vectors peaking at mid-range latitudes (Latitude D) and reaching only 41,000 vectors per year (Fig. 3, right column). This value decreased to 15,000 vectors per year further north and south at Latitudes F and C, and to 1600 at Latitude A.

The duration of the season for the Pulicaris ensemble gradually decreased from Latitude B, where it lasted from week 12 (April) to week 44 (October), toward Latitude F, where it lasted from week 18 (May) to week 41 (October). The vector season was shorter and the abundance lower for the Pulicaris ensemble than the Obsoletus ensemble at all latitude ranges. The mean temperature at the start of the vector season differed between Latitudes A and F. The season started with a mean temperature of 10 °C for the Obsoletus ensemble and 12 °C for the Pulicaris ensemble in southern latitudes, whereas the vector season started at much cooler mean temperatures (1 °C and 3 °C, respectively) at Latitude F (Figs. 2, 3; left columns).

**Fig. 4:**
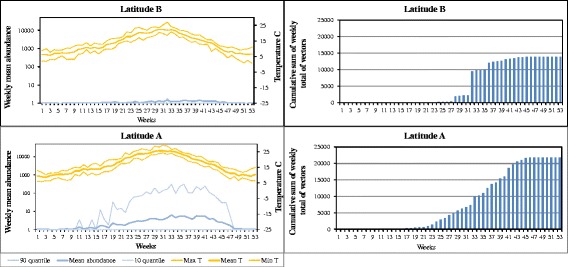
Left column: *C. imicola* weekly average (log scale) with 10th and 90th percentiles for an average year per latitudinal zone (A-B). Right column: number of vectors per year, calculated as the cumulative sum of the weekly average multiplied by 7, by latitudinal range

At Latitude A, the abundance of *C. imicola* increased gradually until it reached the highest mean abundance of nearly 10 vectors per night (Fig. 4). At Latitude B (comprising northern Spain and Corsica), mean abundance was very low (< 3 specimens at the highest peak). This was due to *C. imicola* being almost absent in northern Spain at Latitude B, so the fluctuation of the observed abundance at this latitude was mainly caused by collections made in Corsica. The vector season at Latitude A lasted from week 16 (April) to week 48 (November).

### Abundance and interpolation maps

**Fig. 5:**
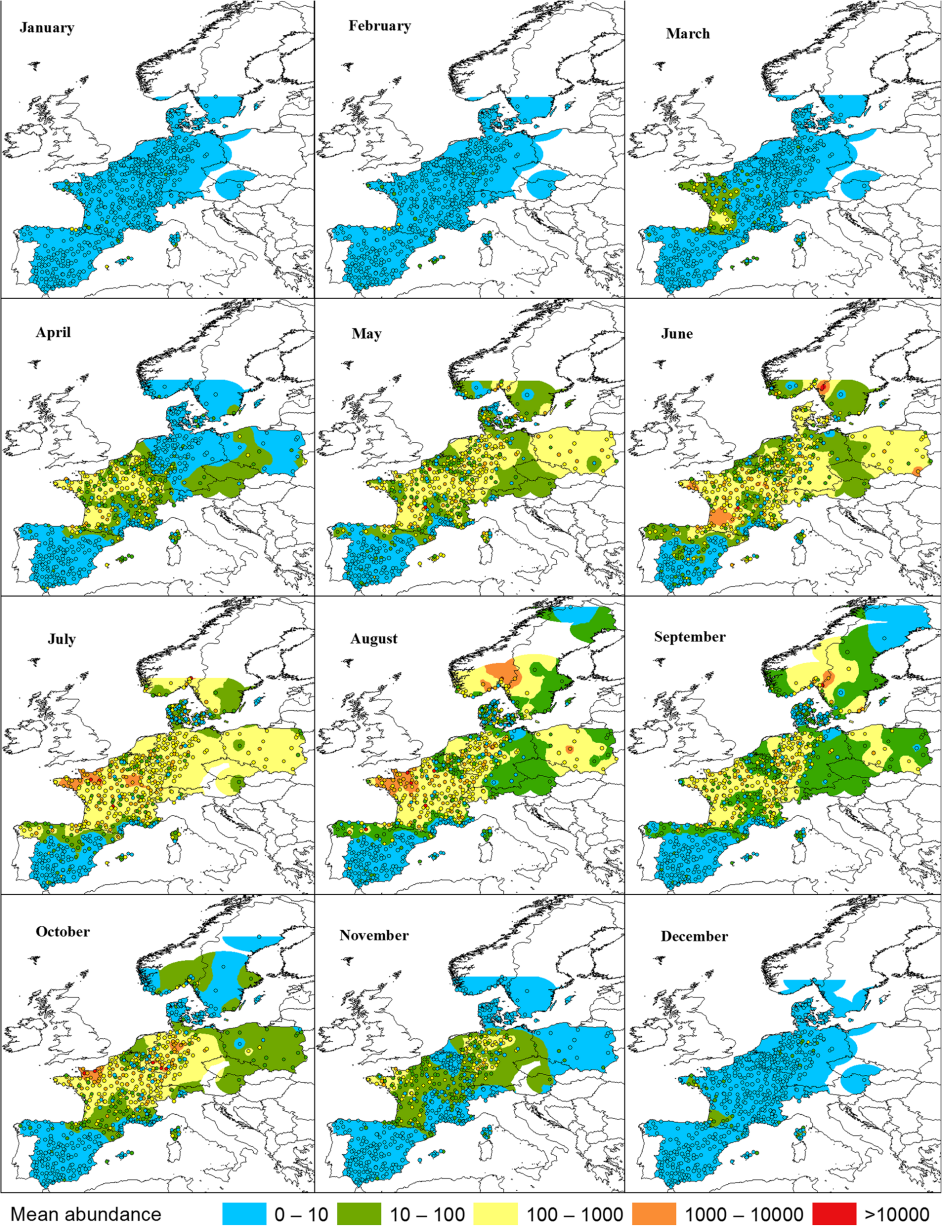
Obsoletus ensemble monthly mean abundance. Dots show observed monthly mean abundance at sampled farms. Spatially interpolated abundance is shown in color. Interpolation values are displayed on the same scale as the observed abundance

**Fig. 6:**
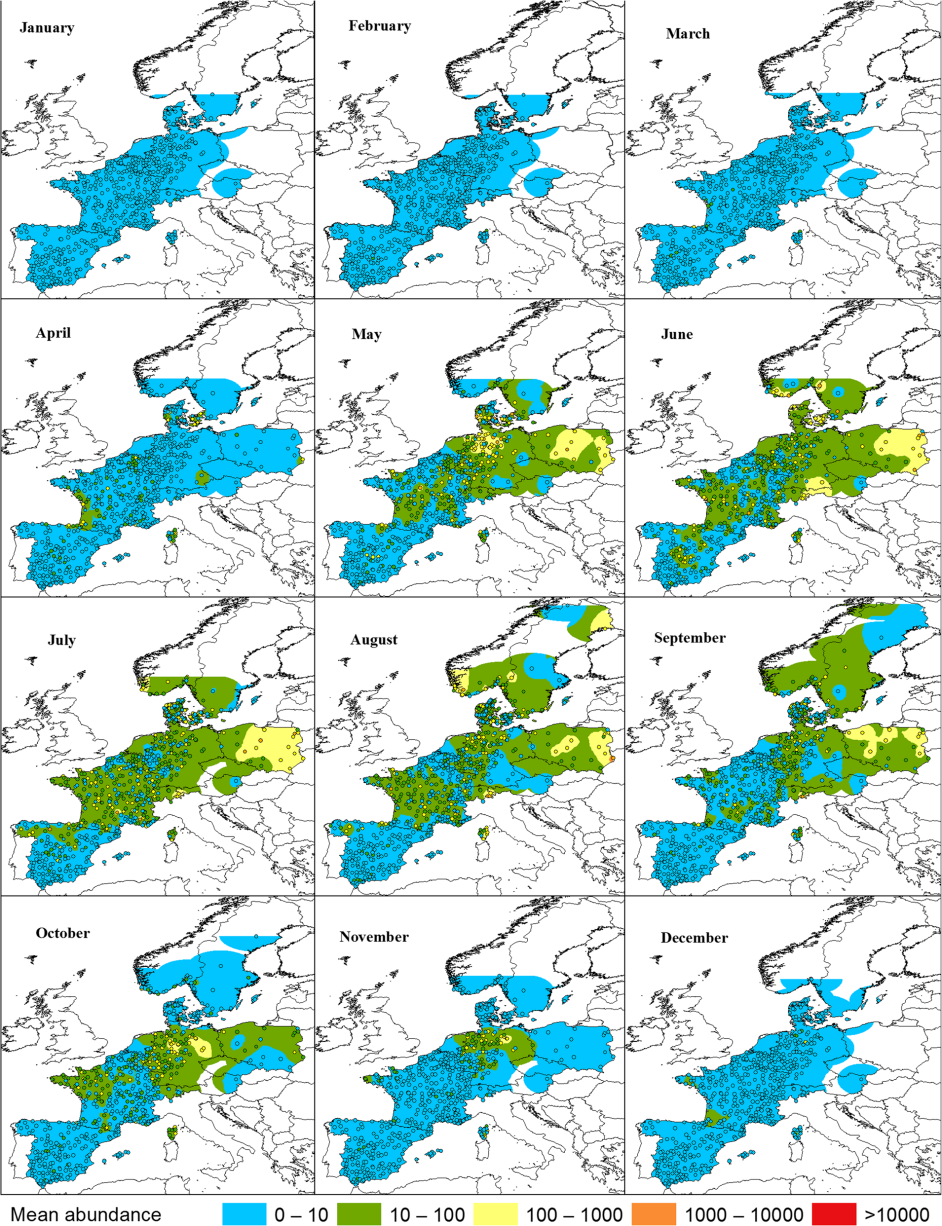
Pulicaris ensemble monthly mean abundance. Dots indicate observed monthly mean abundance in sampled farms. Spatially interpolated abundance is shown in color. Interpolation values are displayed on the same scale as the observed abundance

We observed a large variation in monthly abundance among farms in the same region for all two ensembles and *C. imicola*. However, spatially interpolation the mean monthly abundance at each farm revealed regions with a higher abundance and showed systematic variation within each latitude zone (Figs. 5 and 6). The regions with the highest monthly interpolated abundance of the Obsoletus ensemble were found in France (particularly in the north-west), Germany and southern Scandinavia (especially southern Norway). During the summer period, the monthly interpolated daily abundance was often high (1000–10,000) and occasionally very high (> 10,000) in these countries (Fig. 5). Every month, the interpolated abundance of the Pulicaris ensemble was of a lower magnitude than for the Obsoletus ensemble. During the summer months, farms with low (10–100) and medium (100–1000) abundance were often found distributed throughout the continent (with the exception of Spain), while the regions with the highest abundance were observed in Poland, Germany and Denmark with occasionally high-abundance (1000–10,000) farms (Fig. 6). The distribution in the high abundance regions were therefore found to be in more easterly areas for the Pulicaris ensemble compared to the Obsoletus ensemble.

The geographical areas where the Pulicaris ensemble and the Obsoletus ensemble showed the highest interpolated abundance during the summer months were generally also the areas where the species groups were observed earliest in the spring and latest in the autumn: western France for the Obsoletus ensemble, and Poland and Germany for the Pulicaris ensemble. In regions of low abundance, midges were first observed later and last observed earlier in the year (Figs. 5 and 6).

**Fig. 7:**
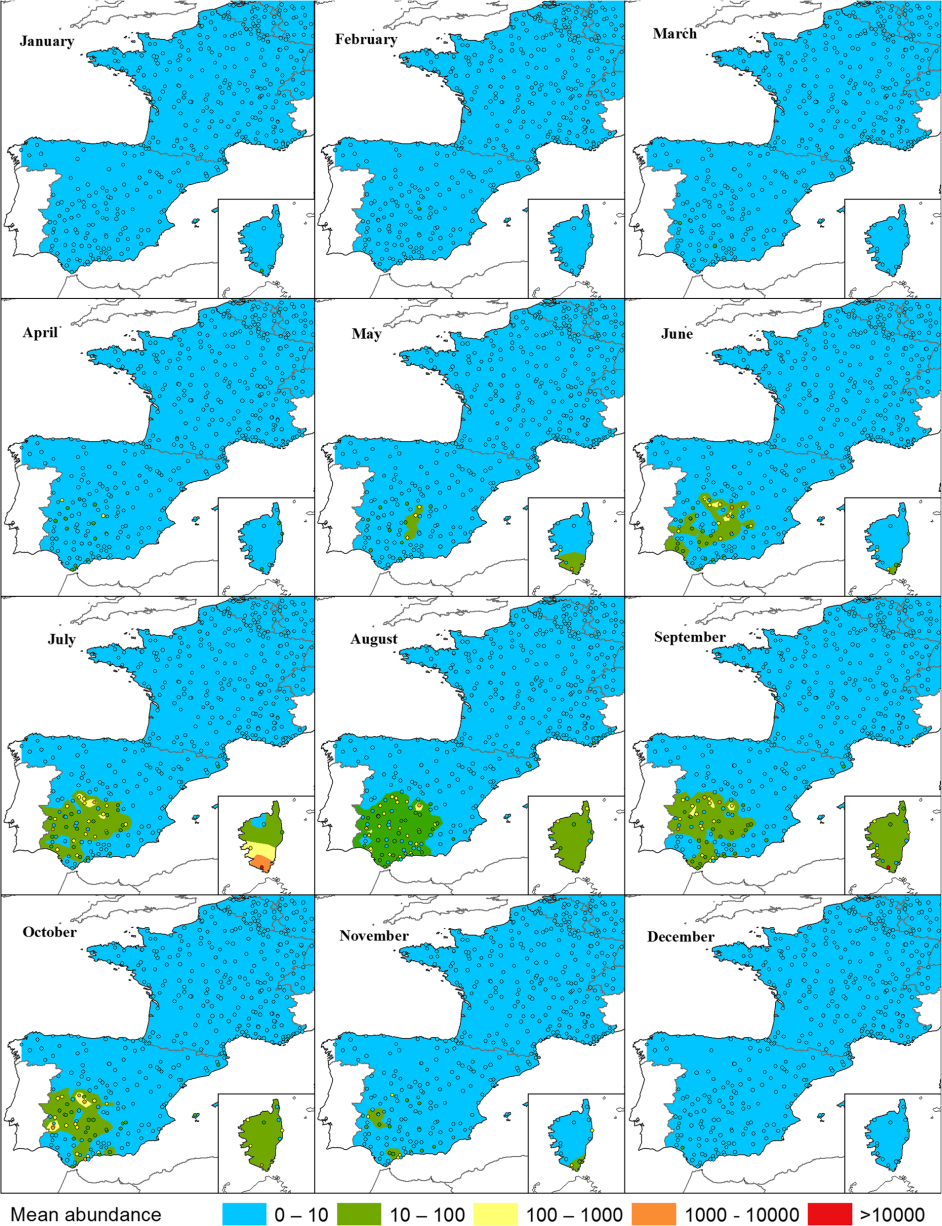
*C. imicola* monthly mean abundance. Dots indicate observed monthly mean abundance in sampled farms. Spatially interpolated abundance is shown in color. Interpolation values are displayed on the same scale as the observed abundance

The highest abundance of *C. imicola* was found in Corsica, where a farm with extremely high abundance was found (> 10,000). In general, Spain had a medium abundance (100–1000), but high-abundance farms could occasionally be found. However, the abundance did not reach the levels seen for the Obsoletus ensemble in northern Europe (Fig. 7).

### Start of vector season by NUTS 3 polygon level

We defined the start of the vector season for each NUTS 3 polygon as the first month with a mean abundance higher than or equal to one specimen for *C. imicola*, and higher than or equal to five specimens for the Obsoletus and Pulicaris ensembles.

**Fig. 8:**
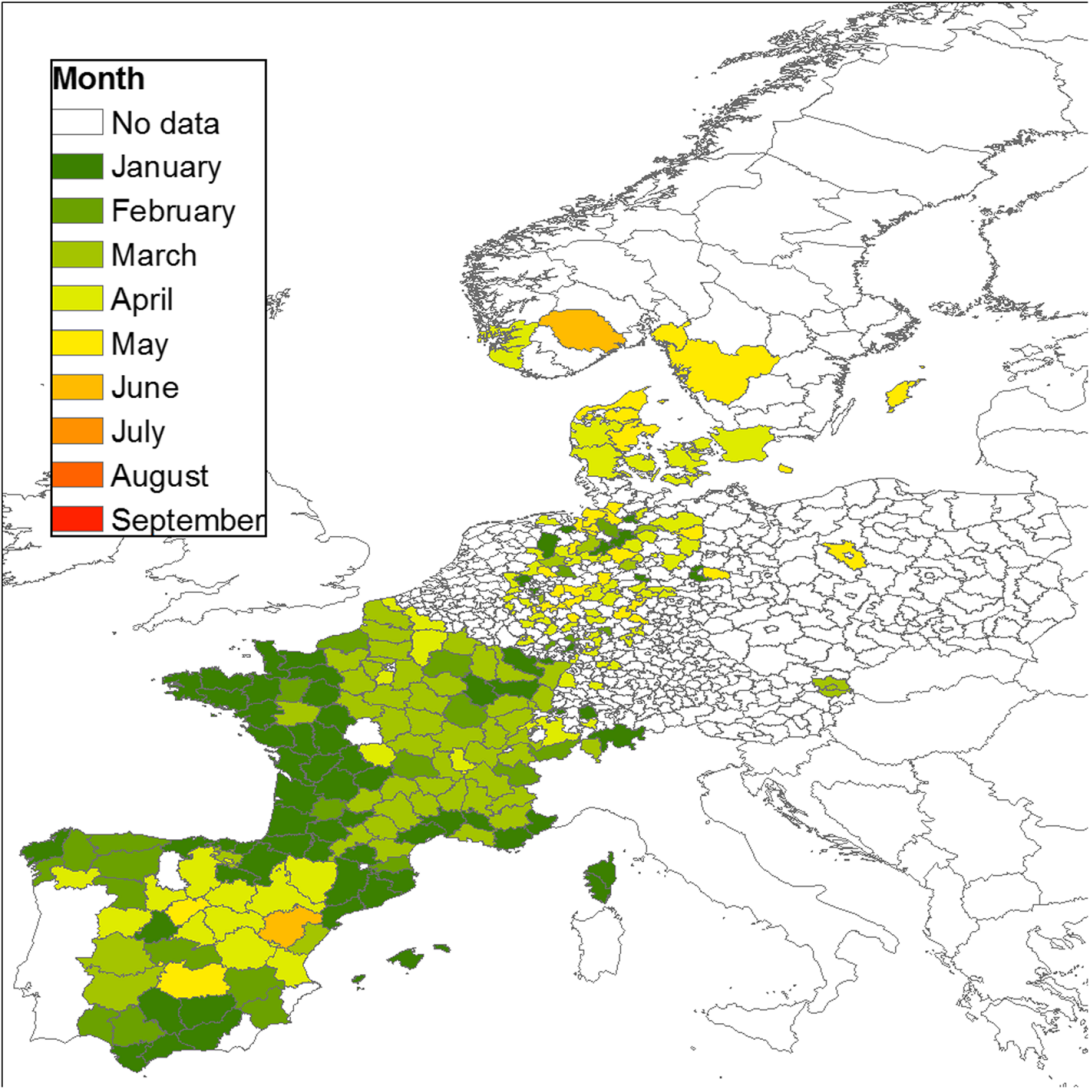
Start of the vector season for the Obsoletus ensemble by NUTS 3 polygons and by month

According to this definition, the start of the season for the Obsoletus ensemble occurred as early as January in the west of France, some parts of Spain and Germany (Fig. 8) and as late as June in Scandinavia. In France, there was a clear spatial pattern where the Obsoletus ensemble season started early (January) in the west, and 2 to 3 months later in the east (March-April). In some provinces in Spain, the season started late (April-June).

**Fig. 9:**
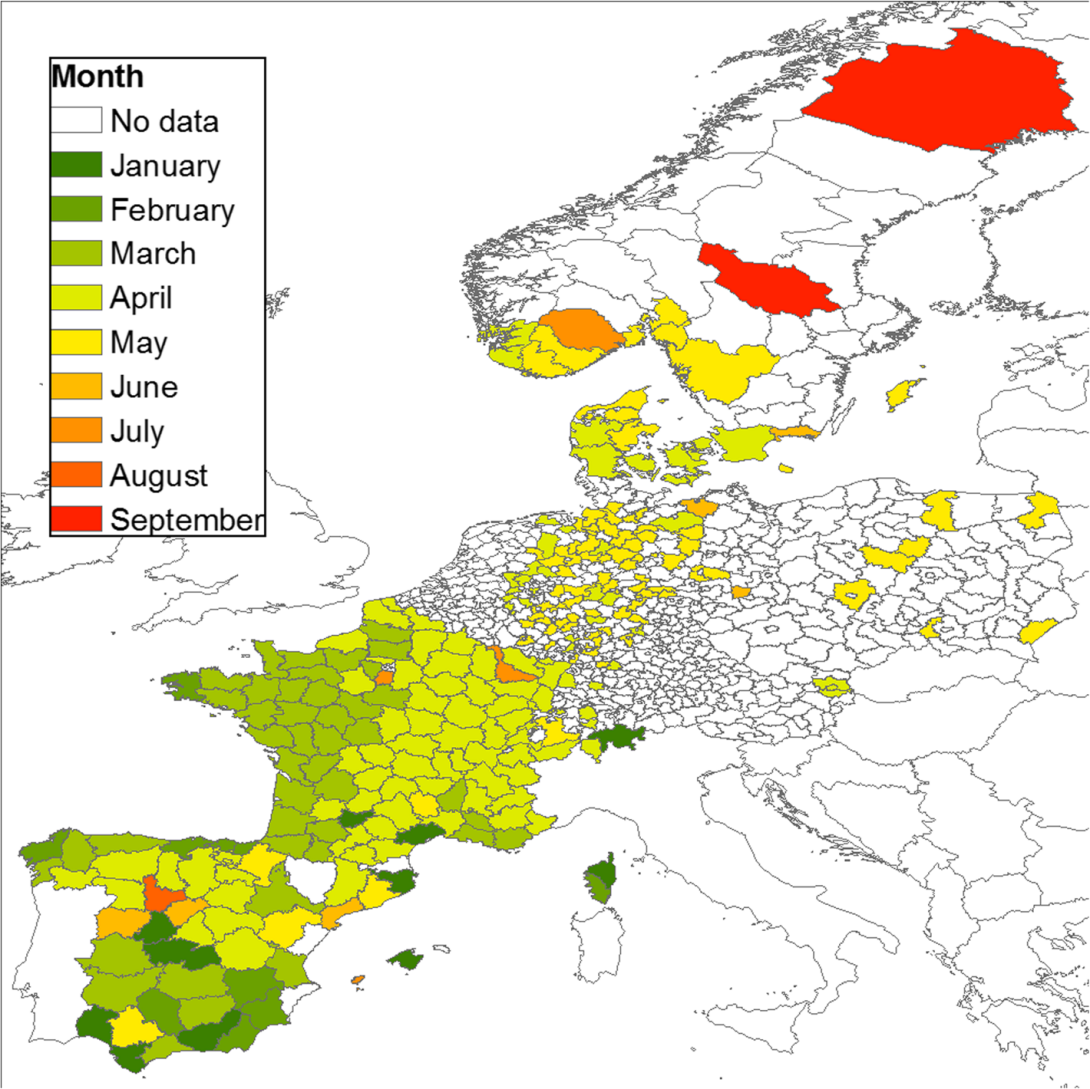
Start of the vector season for the Pulicaris ensemble by NUTS 3 polygons and by month

The start of the vector season for the Pulicaris ensemble showed a spatial pattern similar to the Obsoletus ensemble, with a south-to-north gradient, where southern latitudes had an earlier start of the season (January to April in Spain and France) compared to northern latitudes (May to September in the Scandinavian countries). The start of the season for the Pulicaris ensemble occurred as early as January in some parts of Spain, Germany and Corsica, but generally occurred from March-April, 2 months later than for the Obsoletus ensemble (Fig. 9). In France, we observed the same pattern found for the Obsoletus ensemble, where the start of the season for the Pulicaris ensemble occurred earlier (March) in the west compared to the eastern parts of the country (April).

**Fig. 10:**
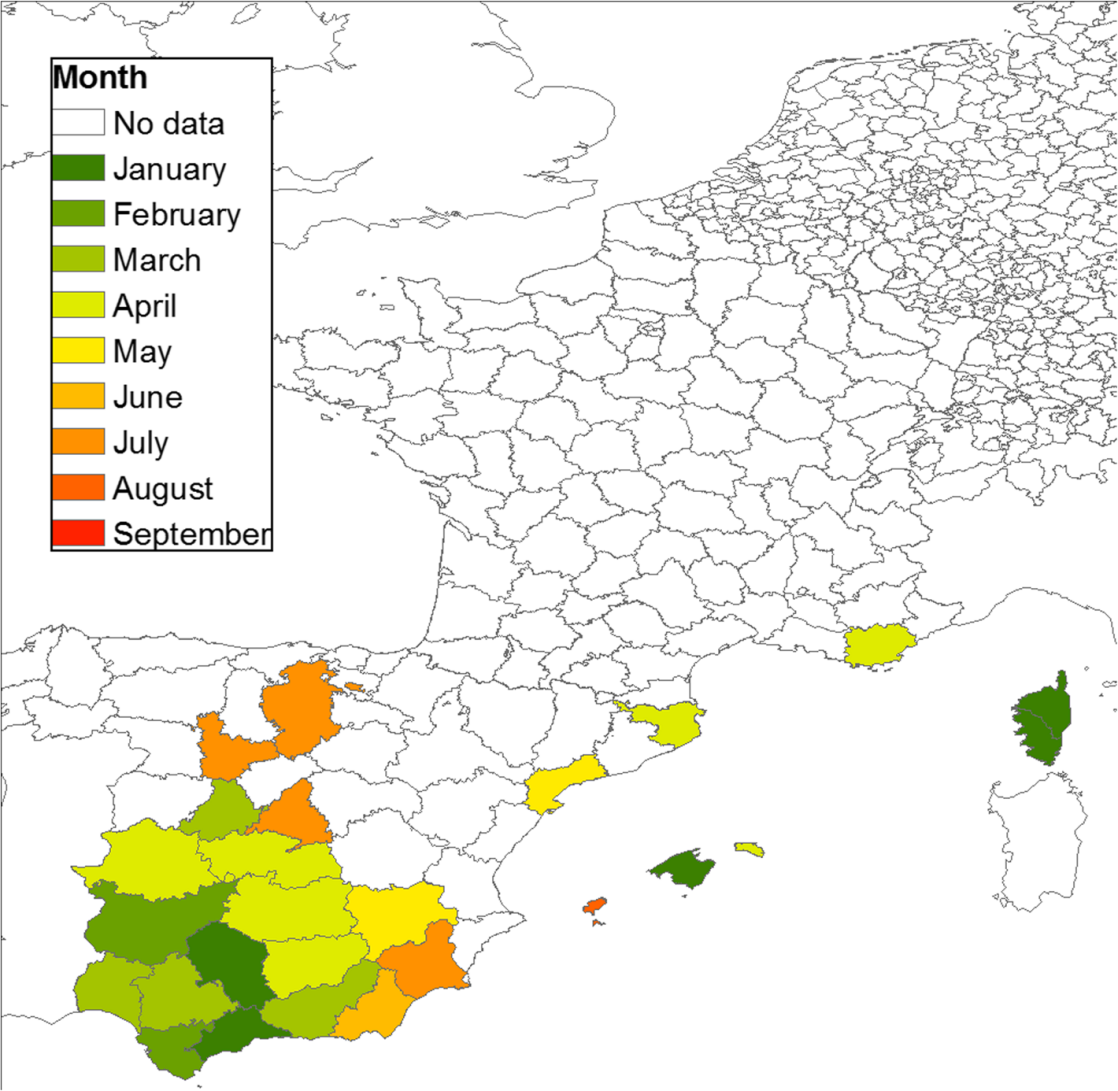
Start of the vector season for *C. imicola* by NUTS 3 polygons and by months

*Culicoides imicola* was only recorded in Spain and France. The vector season started as early as January in southern Spain and on Corsica, while in the northern provinces of Spain, it started 5 months later (Fig. 10). However, there were also two provinces in the south of Spain where the average abundance per polygon did not reach the threshold value of one until June-August.

## Discussion

The descriptive analysis presented here is based on the most extensive *Culicoides* dataset created for Europe to date, and represents the first combined description of *Culicoides* abundance and distribution for a large part of Europe. The data were gathered from a 4000 km long transect from southern Spain to the Arctic Circle in Sweden, with the most easterly collection sites in Poland. The primary aim of this descriptive analysis was to identify and quantify major geographical patterns and seasonal trends in the abundance of key *Culicoides* vector groups. The focus of the analysis was to identify patterns and trends important for decision making to prevent, surveillance and control of *Culicoides*-borne pathogens.

Specimens of the Obsoletus and Pulicaris ensemble were found in all of the countries sampled. The Obsoletus ensemble was ten times more abundant than the Pulicaris ensemble. Both groups have a Palaearctic distribution, and are widely distributed in Europe [13, 44–47]. However, the abundance of both ensembles and *C. imicola* varied dramatically among farms in the same region sampled during the same period, often showing a 100-fold difference between the 10th and the 90th percentile in terms of the weekly trap abundance within each latitude group. Nevertheless, distinct spatial and temporal patterns arose under the three analyses conducted in this work.

Examining the weekly data for the seven latitude ranges, we found that the mean weekly abundance of both ensembles and *C. imicola* varied dramatically along the south-north transect. It is interesting to note that the annual number of the Obsoletus ensemble gradually increased toward northern latitudes, despite the vector season gradually shortening by three months from southern Spain to mid Scandinavia. This suggests that the Obsoletus ensemble is better adapted to the northern European climate in the environments surrounding farms. The Pulicaris ensemble appear better adapted to central Europe, and *C. imicola* to southern Europe, in the dry Mediterranean regions characterized by hot summers [47]. Although in relative terms, the Pulicaris ensemble (*C. pulicaris* and *C. punctatus*) was more abundant in central Europe and the Obsoletus ensemble (composed of *C. obsoletus*, *C. scoticus*, *C. montanus* and *C. chiopterus*.) was more abundant in northern Europe, the Obsoletus ensemble was still more abundant than the Pulicaris ensemble at all latitudes.

To further explore the spatial abundance patterns, we interpolated the mean monthly abundance for each calendar month. We found areas of high abundance peaks for the Obsoletus ensemble in north-western France, which is in agreement with previous studies that also reported high numbers of Obsoletus ensemble specimens in France [9, 22, 23]. The Obsoletus ensemble was also found in high abundance in most parts of Germany, where similar findings have been reported by many other authors [14, 19, 24, 47, 48]. Regarding the high-abundance areas found in Scandinavia in this study, these were partly driven by farms with an extremely high abundance; for example, two farms in southern Norway had more than 80,000 specimens per night (data not shown). Although the monthly mean data were log_10_ transformed before interpolation in order to reduce the impact of single sites of very high abundance, these high abundance farms from Norway still influenced the observed regional pattern. These high abundance records exceeded in great magnitude, the *C. obsoletus*/*C. scoticus* abundance previously reported in Sweden by Ander et al. [33] (> 5000 specimens in suitable months). Further collections in southern Norway are needed to determine how often the Obsoletus ensemble occurs at these extremely high numbers. The traps with the highest abundance of the Pulicaris ensemble were located in Germany and Poland, with a lower abundance in France and Scandinavia [14, 49, 50]. In general, the Pulicaris ensemble showed a spatial pattern with a relatively more easterly distribution compared to the Obsoletus ensemble. The distribution of the regions with the highest *C. imicola* abundance were in accordance with the known *C. imicola* distribution in Europe [9, 21, 22, 47]. Based on the interpolated monthly abundance maps, each vector group tended to appear early in the traps situated in areas that reached the highest abundance during summer, and were observed latest in the autumn. When the monthly maps are considered as a time series, the Obsoletus ensemble appears to start in western France, to increase in abundance and spread north and east until the end of August, when they retract to western France again. The Pulicaris ensemble appears to start in traps in Poland and, to a certain extent, the south-western areas of France, to grow in number and spread north and east before retracting again after August, to be observed last in Poland, northern Germany and western France. For *C. imicola*, the same phenomenon of areas with a strong spatial correlation between early appearance, peak abundance and a long vector season was seen in southern Corsica and south-eastern Spain.

The *Culicoides* abundance analysed in this study is exclusively based on the abundance observed on farms. The spatial abundance derived by interpolation therefore represents the abundance given the presence of a farm, and cannot be interpreted as an estimate of abundance in “non-farm” habitats, e.g. natural areas. Interpolation is used as tool to visualize the average abundance on farms in a larger area. To produce more detailed maps of vector abundance in future, a predictive modelling approach based on environmental and climate predictor variables will be necessary.

We analysed the start of the vector season for polygon areas in the participating countries. The European Commission currently defines the start of the vector season as the week during which the number of parous females exceeding a certain threshold (five for *C. obsoletus* and one for *C. imicola*) are caught by any trap in an area [20]. However, because there is a large variation in abundance among traps in the same area, the probability of finding a trap with an abundance exceeding the threshold will increase with the number of traps operated in the area. As a result, the vector season will tend to start earlier in regions with a higher number of traps. In this analysis, where the density of traps varies geographically, we used a more robust approach to define the start of the vector season. We took into account the number of traps in a geographical area by calculating the mean trap abundance and using five vectors per trap as the cutoff (one female per trap for *C. imicola*). For the Obsoletus ensemble, there was not a clear pattern in Spain, where the vector season started at different months in different polygons. Nevertheless, there was a south-north trend starting in southern France and continuing to northern Scandinavia. The length of the vector season decreased by three months at northern latitudes where the period of climatic conditions suitable for midge development is shorter. Versteirt et al. [51] investigated the start and end of the vector season for *C. imicola* and *C. obsoletus*/*C. scoticus* on a continental scale in Europe. Their results also showed a south-north pattern in the start and length of the vector season. However, for *C. imicola*, our observed data suggested that the start of the season would occur earlier in the year (January–February) compared to their results where the season started in March. The start of the Pulicaris ensemble season had a similar pattern to the Obsoletus ensemble, starting two months later at higher latitudes; however, the start of the season seems to be more homogenous across latitudes compared to Obsoletus ensemble. We found the start of the season pattern for *C. imicola* is similar to previous results [51], with the only difference that in our study the vector season started as early as January on Corsica and in some provinces of Spain, which we interpret as areas with *C. imicola* vector activity all year round. The start of the season occurred later in the year in polygons where the peak *Culicoides* abundance in summer was low, as the density gradually increased and the cutoff defining the start of the vector season was reached later than in areas with a high peak abundance. For example, the Pulicaris ensemble activity started as late as September in northern Sweden, despite the first individual being observed much earlier. The start of the vector season is therefore highly sensitive to the vector abundance threshold selected to define it.

The latitude range analysis showed that the vector season for both the Obsoletus and Pulicaris ensembles started in the southern latitude range at an average mean temperature of 10 °C and 12 °C, respectively. However, at northern latitudes, the season started before spring temperatures rose, meaning that the vector season started at mean temperatures of just 1 °C and 3 °C. This suggests an adaptation of the vector population to the cooler climates in northern Europe. Early EU regulations suggested that vector-free periods may be defined by a specific temperature threshold when vector surveillance data were lacking [46]. Yet the start of the vector season at successively lower temperatures toward northern latitudes found in the present study demonstrates that such a simple temperature criterion alone would be a poor proxy for the start of the vector season, and would risk the prediction of an unrealistically long vector-free season in northern Europe.

One country used CDC traps (Spain) and one used BG-sentinel traps (Germany), while the rest used Onderstepoort traps. Previous studies compared the efficacy of different trap types, and the results showed that the number of midges collected varied according to the type used. It was reported that Onderstepoort traps collected more specimens than Mini CDC and BG-sentinel traps [35–37]. We attempted to adjust for this using published trap comparisons, but the relative efficiency of each type trap vary considerably. However, this uncertainty in trap efficiency is likely to be of a smaller magnitude than the variation in abundance within the spatial patterns which is of at least a 10 to 100 fold magnitude. The used trap conversion factor is therefore unlikely to have affected the identified overall spatial patterns identified here.

Light traps are most efficient when collection nights are dark [10, 52] and trapping in northern latitudes may therefore be less effective because nights during the summer season are shorter than in southern Europe [53]. The use of light traps in this study may therefore have underestimated the vector abundance at higher latitudes during the summer period.

We here focus on estimating the host seeking vector population. The vectors collected in light traps near stables and animal resting sites are likely to be predominantly host-seeking, while vectors that are already blood-fed are less likely to be collected in traps. The results from this analysis cannot directly be used for estimating the total vector population consisting of both host seeking and non-host seeking vectors. Because the blood meal digestion time in *Culicoides* is relatively slower at low temperatures [54], the proportion of the *Culicoides* population digesting blood and developing eggs and not attracted to traps may therefore be relatively larger at low temperatures. Due to the increasingly lower temperatures toward the north of the transect, the total vector population estimated from trap collections at higher latitudes, will be underestimated compared to the total vector population estimated from trap collections in southern Europe unless the blood meal developing time is taken into account.

Females of the species belonging to the Obsoletus group are difficult to separate based on morphological characters [13, 18, 38–41, 55] and therefore they are often grouped into the Obsoletus group or complex. The same occurs with the species of the Pulicaris group which are often merged into the Pulicaris group [25, 31, 56, 57]. Aggregating species into groups might represent a problem for identifying accurate temporal and spatial patterns, as different species from the same group might exhibit different seasonal trends [58, 59]. The seasonal fluctuation of individual species of the Obsoletus group remains unknown. However, Searle et al. [27] analysed the phenology (start, end and duration of the vector season) of the male specimens for each species and the authors did not find a significant difference between the start and the end of season among the species. Nevertheless they found the length of the season period was different among them. Analyses regarding temporal fluctuation at species level of the Obsoletus group would be necessary as the species might present different temporal fluctuation patterns undetected when the species are grouped. Little is known about potential differences in vector competence for BTV for individual species of the Obsoletus and Pulicaris ensemble [11]. A study on the subject includes Carpenter et al. [60] who analysed vector competence for the Obsoletus group in the UK. Vector competence at species level of the Obsoletus group for SBV can be found in [60], Balenghien et al. [61] and Ségard et al. [62]. In this work, despite analyzing the data at ensemble level, we consider that the identified patterns and trends identified here still represent a useful and relevant overview of transmission potential in Europe.

## Conclusions

This is the first report in which a dataset this size and covering a large part of Europe has been analysed. We identified and quantified the main mean spatial and temporal differences of three Culicoides species groups. Understanding the spatial and seasonal patterns of key vector groups or species facilitates the planning of preventive strategies and allows the development of more cost-effective vector and disease surveillance programmes by veterinary authorities in the European Union. The monthly abundance of the Obsoletus ensemble increased gradually from northern Spain to mid Scandinavia. The vector season also became increasingly shorter toward the north, starting three months later in mid Scandinavia compared to southern Spain. Nevertheless, the annual accumulated abundance of the Obsoletus ensemble increased steadily with latitude to 500,000 vectors per trap per year in mid Scandinavia. The Pulicaris ensemble was more frequent in central Europe, peaking in Germany and Poland with about 40,000 vectors per year, and with a more easterly distribution compared to the Obsoletus ensemble. For each of the species groups, there were areas in which the vectors appeared early, reached the highest mean peak abundances and lasted the longest. The Obsoletus ensemble was more abundant and had a longer season than the Pulicaris ensemble, whereas *C. imicola* appeared as a strictly southern species with a long vector season but with an abundance level that did not reach the peak abundance observed for the Obsoletus ensemble. This study suggests that future collaboration and data sharing between European countries may further improve our understanding of the spatio-temporal abundance of *Culicoides* vectors.

## Additional file

Additional file 1: Table S1. Monthly availability of *Culicoides* trap data in the participating countries during the selected seven-year study period (2007–2013). X symbol indicates months when data were available. (XLSX 12 kb)

## Abbreviations

BTV: bluetongue virus
NUTS: Nomenclature of territorial units for statistics
SBV: Schmallenberg virus
